# Associations of Prestroke Physical Activity With Stroke Severity and Mortality After Intracerebral Hemorrhage Compared With Ischemic Stroke

**DOI:** 10.1212/WNL.0000000000201097

**Published:** 2022-11-08

**Authors:** Adam Viktorisson, Dongni Buvarp, Malin Reinholdsson, Anna Danielsson, Annie Palstam, Katharina Stibrant Sunnerhagen

**Affiliations:** From the Institute of Neuroscience and Physiology (A.V., D.B., M.R., A.D., A.P., K.S.S.), Rehabilitation Medicine, Sahlgrenska Academy, University of Gothenburg, and the Sahlgrenska University Hospital; Department of Occupational Therapy and Physiotherapy (M.R.), Sahlgrenska University Hospital; Institute of Neuroscience and Physiology (A.D.), Department of Health and Rehabilitation, Sahlgrenska Academy, University of Gothenburg; and School of Health and Welfare (A.P.), Dalarna University, Falun, Sweden.

## Abstract

**Background and Objectives:**

Prestroke physical activity may protect the brain from severe consequences of stroke. However, previous studies on this subject included mainly ischemic stroke cases, and the association between prestroke physical activity and outcomes after intracerebral hemorrhage is uncertain. Therefore, we sought to examine the associations between prestroke physical activity, stroke severity, and all-cause mortality after intracerebral hemorrhage in comparison with ischemic stroke.

**Methods:**

This was a longitudinal, register-based cohort study. All adult patients with intracerebral hemorrhage or ischemic stroke admitted to 3 stroke units in Gothenburg, Sweden, between November 1, 2014, and June 30, 2019, were screened for inclusion. Physical activity was defined as light physical activity ≥4 h/wk or moderate physical activity ≥2 h/wk the year before stroke. Stroke severity was assessed on admission using the NIH Stroke Scale. All-cause mortality rates were followed up to 7 years, from the time of incident stroke until death or censoring. Ordinal logit models and Cox proportional hazards models were used to estimate adjusted associations of prestroke physical activity.

**Results:**

We included 763 patients with intracerebral hemorrhage and 4,425 with ischemic stroke. Prestroke physical activity was associated with less severe strokes by an adjusted odds ratio of 3.57 (99% CI 2.35–5.47) for intracerebral hemorrhages and 1.92 (99% CI 1.59–2.33) for ischemic strokes. During a median follow-up of 4.7 (interquartile range 3.5–5.9) years, 48.5% of patients with intracerebral hemorrhage died, compared with 37.5% with ischemic stroke. Prestroke physical activity was associated with decreased short-term mortality (0–30 days) by an adjusted hazard ratio of 0.30 (99% CI 0.17–0.54) after intracerebral hemorrhage and 0.22 (99% CI 0.13–0.37) after ischemic stroke. Prestroke physical activity was further associated with decreased long-term mortality (30 days–2 years) by an adjusted hazard ratio of 0.40 (99% CI 0.21–0.77) after intracerebral hemorrhage and 0.49 (99% CI 0.38–0.62) after ischemic stroke.

**Discussion:**

Prestroke physical activity was associated with decreased stroke severity and all-cause mortality after intracerebral hemorrhage and ischemic stroke, independent of other risk factors. Based on current knowledge, health care professionals should promote physical activity as part of primary stroke prevention.

The health benefits of physical activity are significant, and there is a dose-response relationship between the volume of physical activity and risk of cardiovascular morbidity and mortality.^1,2^ Regular physical activity serves as an important component in the primary and secondary prevention of stroke^3,4^ and counteracts several cerebrovascular risk factors.^5,6^ However, the association between prestroke physical activity and outcomes after stroke is not well established. Prestroke physical activity has been associated with reduced stroke severity,^7-12^ in-hospital mortality,^12^ cardiovascular mortality,^13^ and all-cause mortality.^13-16^ Contrarily, some previous studies found no association between prestroke physical activity and stroke severity or poststroke mortality, after covariate adjustment.^17-19^ Moreover, the relationship between prestroke physical activity and poststroke outcomes has been studied only in samples with most ischemic stroke cases. No previous study has investigated the association between prestroke physical activity and stroke severity or the risk of mortality for intracerebral hemorrhage.^20^

Nontraumatic intracerebral hemorrhage is a spontaneous bleeding within the brain parenchyma and usually a long-term consequence of arterial hypertension or cerebral amyloid angiopathy.^21^ It is the most lethal type of stroke, with early mortality rates greater than 40% and no trend toward improvement in recent decades.^22^ The global socioeconomic consequences of intracerebral hemorrhages are significant, and the incidence is particularly high in low-income populations.^23^ Modifiable predictors of outcomes after intracerebral hemorrhage are scarce, and there is no largely applicable intervention that can improve the outcome for those affected.^24^ Regular physical activity decreases the risk of intracerebral hemorrhage,^25^ prevents small vessel disease through decreased arterial blood pressure,^26^ and could potentially lower levels of circulating beta-amyloid.^27^ Recent preclinical data also suggest that preconditioning physical activity reduces hematoma volume and facilitates recovery after induced intracerebral hemorrhage in mice.^28^

The physical activity prestroke in Gothenburg (PAPSIGOT) is a register-based project that investigates the association between physical activity and outcomes after stroke (Swedish registry of trials number: 246671). In this study, we investigated the associations between prestroke physical activity, stroke severity, and all-cause mortality in patients with intracerebral hemorrhage compared with patients with ischemic stroke.

## Methods

### Standard Protocol Approvals, Registrations, and Patient Consents

This study was approved by the Regional Ethical Board of Gothenburg on May 4, 2016 (registration number: 346-16) and by the Swedish Ethical Review Authority in 2021 (registration number: 2021-03324). Research using quality registers (Väststroke and Riksstroke) are exempt from the general rule of patient consent according to the Swedish Personal Data Act (Swedish law No. SFS 1998:204), but patients are informed about the possibility to opt out from the registers. The Swedish Personal Data Act also allows data from medical records to be collected for quality control, without receiving written informed consent.

### Data Collection

In this longitudinal, register-based cohort study, we collected data on all adult patients with intracerebral hemorrhage admitted to 3 stroke units between November 1, 2014, and June 30, 2019. We also included all adult patients with ischemic stroke treated during this period, with complete data on corresponding variables to patients with intracerebral hemorrhage. Stroke cases were identified in the Väststroke register, which includes data from routine clinical work on all patients treated for stroke within the Sahlgrenska University Hospital in Gothenburg, Sweden. Identified stroke cases were cross-referenced with inpatient medical records to ensure reliability and reduce missing data. We reviewed and included assessments of stroke characteristics, physical activity, smoking status, and comorbidities diagnosed on hospital arrival. Additional stroke characteristics were collected from the National Swedish Stroke Register (Riksstroke). Socioeconomic variables were collected from the Swedish Longitudinal Integrated Database for Health Insurance and Labor Market Studies. Prestroke comorbidities were collected from the National Patient Registry. Survival rates were collected from the Swedish Cause of the Death Register. Data merging with national registries were performed by the National Board of Health and Welfare using the personal identity numbers that are provided to all residents in Sweden at birth or shortly after immigration. Patients with a nonspecified stroke (i.e., no radiology), unavailable medical records, or lacking a Swedish personal identity number were excluded. A follow-up of poststroke survival continued until October 19, 2021. The study adheres to the REporting of studies Conducted using Observational Routinely collected health Data statement.^29^

### Outcomes

Admission stroke severity was assessed using the NIH Stroke Scale (NIHSS).^30^ Higher NIHSS scores indicate greater stroke severity, with a maximum score of 42. The NIHSS scores were categorized as severe (>14), moderate (5–14), or mild (0–4). The NIHSS scores were assessed using medical records for cases with missing data, based on the first neurologic examination at the hospital, before any documented intervention.^31^ Patients in a comatose state, for which a complete neurologic examination was not possible, were categorized as having a severe stroke. All-cause mortality and cerebrovascular disease (CVD) as underlying causes of death were recorded for all included patients. CVD was defined according to the International Statistical Classification of Diseases and Related Health Problems, Tenth Revision codes (I60–I69). The follow-up time for survival was measured from the time of incident stroke until death or censoring on October 19, 2021, allowing for a minimum follow-up of >2 years. Short-term mortality was defined as 0–30 days and long-term mortality as 30 days to 2 years.

### Prestroke Physical Activity

Physical activity was defined as light physical activity ≥4 h/wk or moderate physical activity ≥2 h/wk during the year before stroke, using the Saltin-Grimby Physical Activity Level Scale (SGPALS).^32,33^ Light physical activity includes activities such as bicycling, walking, gardening, or bowling, in the SGPALS; whereas moderate physical activity includes activities such as running, swimming, gymnastics, or playing tennis. The assessments were performed by physiotherapists working at the included stroke units. Information on prestroke physical activity was collected from a next of kin if the patient had impaired cognition or was unconscious. For cases with missing data on SGPALS in the Väststroke register, assessments were based on available information from medical records. Patients who were unable to move freely indoors before the stroke were always categorized as inactive.

### Definition of Covariates

All covariates were recorded during incident stroke and included sex, age, country of birth, education, income, living situation, independence in activities of daily living, smoking, alcohol disorder, drug abuse, comorbidities, and prestroke medical treatments. Income was defined as household income during the year before the stroke and congregated in tertiles. Educational level was defined as the highest completed stage, trichotomized in <10 years (primary school), 10–12 years (secondary school), and >12 years (postsecondary or university education). Smoking was defined as current smoking or absence of smoking for <1 year. If smoking status was not recorded in the registries or medical records, the patient was considered a nonsmoker. Prestroke comorbidities and medications accounted for are listed in eTables 1 and 2 (links.lww.com/WNL/C265).

### The Charlson Comorbidity Index

The Charlson comorbidity index was calculated for each patient during incident stroke.^34^ It is a weighted score validated to predict the risk of 1-year mortality for hospitalized patients, based on specific comorbid conditions. Survival rates were stratified using Charlson comorbidity index scores in groups with no comorbidity (0 points), mild comorbidity (1–2 points), and severe comorbidity (>2 points).

### Statistical Analysis

A priori power calculation was performed to estimate the required period for data collection and has been previously published.^35^ The calculation was based on NIHSS scores trichotomized in 3 groups.^10^ We calculated that a sample size of >628 patients with intracerebral hemorrhage would be sufficient for analyses at an alpha level of 0.01 with 0.8 power. Descriptive statistics for patients with missing observations are summarized in eTable 3 (links.lww.com/WNL/C265). Missing observations were handled using predictive mean matching, implemented for multiple imputation by chained equations.^36^ Each variable was imputed through an iterative series of predictive models for ordered categorical data. All covariates and outcome variables were included in each model. However, separate imputation models were used for patients with intracerebral hemorrhage and ischemic stroke.

Descriptive data are presented as counts and proportions for categorical variables or median with interquartile range (IQR) for ordinal variables. Multicollinearity was assessed using the Spearman rank correlation for ordinal variables and the Phi coefficient for nominal variables. Adjusted associations between prestroke physical activity and admission stroke severity (NIHSS scores 0–4 vs 5–14 or >14) were evaluated using ordinal logit regression models for intracerebral hemorrhage and ischemic stroke, respectively. Only variables with ≥10 observations in each outcome category were included. Cumulative survival probabilities were calculated with additional Kaplan-Meier curves. Associations between prestroke physical activity and short-term and long-term all-cause mortality was evaluated using Cox proportional hazards models. Schoenfeld residuals were used to check the independence between residuals and time.

All statistical tests were interpreted at a 2-tailed significance level of 0.01 to account for multiple comparisons. Adjusted odds ratios (aORs) and adjusted hazard ratios (aHRs) for included covariates were derived from the respective model coefficients, and accompanying 99% CIs were presented for each covariate. There was no stepwise selection in the regression models because all included covariates were considered potential confounders to the association between prestroke physical activity and stroke outcomes. All analyses were performed using SPSS Statistics (IBM Corp. IBM SPSS Statistics for Windows, Version 27.0. Armonk, NY) and R software (R Core Team, Version 4.0.2 for Windows; R Foundation for Statistical Computing, Vienna, Austria).

### Data Availability

Anonymized data not published within this article will be made available to any qualified investigator by Professor Katharina Stibrant Sunnerhagen.

## Results

### Patient Characteristics

During the study period, 770 patients were diagnosed with intracerebral hemorrhage. In total, 6 patients were excluded because they lacked a Swedish personal identity number and 1 because of unavailable medical records. The final sample consisted of 763 patients with intracerebral hemorrhage in addition to 4,425 patients with ischemic stroke. Baseline characteristics of the study population are summarized in Table 1. Among physically active patients with intracerebral hemorrhage, 255 (88.5%) performed light physical activity for ≥4 h/wk and 33 (11.5%) moderate physical activity for ≥2 h/wk the year before stroke, compared with 1,659 (86.8%) and 252 (13.2%), respectively, among patients with ischemic stroke. The localizations of hematomas were similarly distributed in physically active and inactive patients; however, only inactive patients presented with primary intraventricular hemorrhages (eTable 4, links.lww.com/WNL/C265). After intracerebral hemorrhage, 50 (6.6%) patients underwent neurosurgery, while 7 patients with ischemic stroke were treated with hemicraniectomy, 381 (8.6%) were treated with thrombectomy, and 558 (12.6%) received thrombolysis.

**Table 1:**
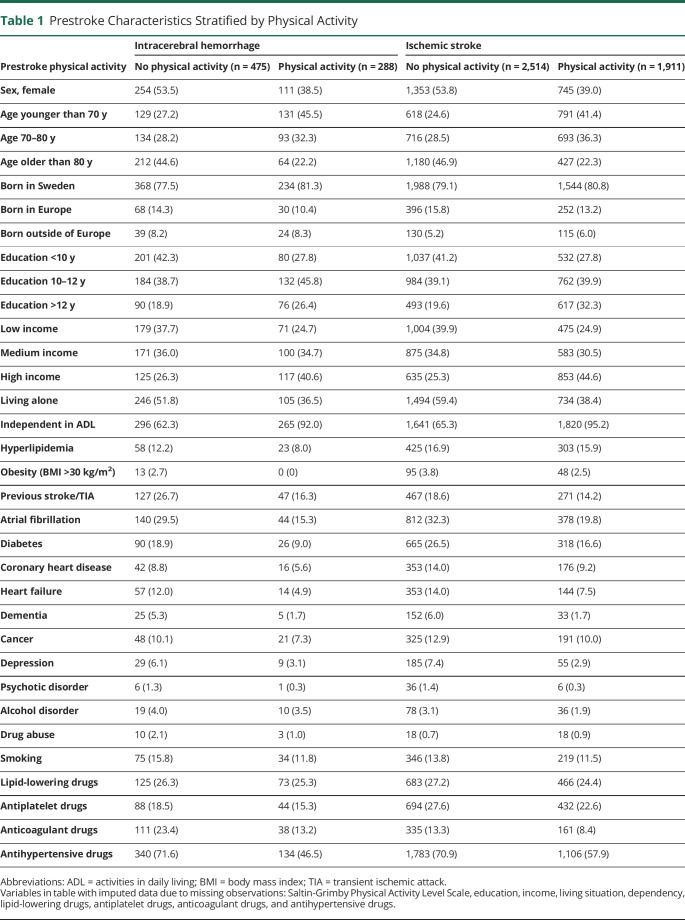
Prestroke Characteristics Stratified by Physical Activity

### Stroke Severity

**Figure 1:**
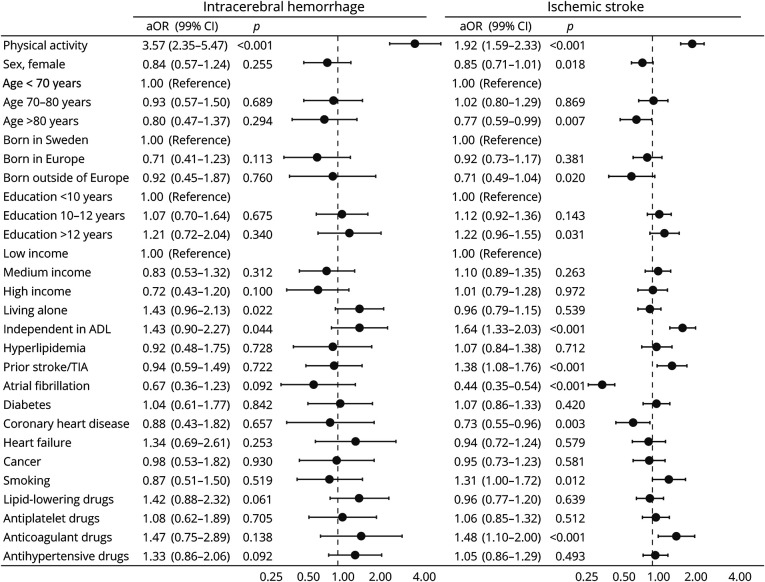
Associations Between Covariates and Lower Stroke Severity Multivariate ordinal logit models predicting less severe stroke symptoms (NIHSS scores 0–4 vs 5–14 or >14) for patients with intracerebral hemorrhage and ischemic stroke. aOR = adjusted odds ratio; NIHSS = NIH Stroke Scale; TIA = transient ischemic attack. Adjusted associations from models including prestroke physical activity, sex, age, country of birth, education, income, living situation, dependency, hyperlipidemia, previous stroke/TIA, atrial fibrillation, diabetes, coronary heart disease, heart failure, cancer, smoking, lipid-lowering drugs, antiplatelet drugs, anticoagulant drugs, and antihypertensive drugs.

In the group of patients with intracerebral hemorrhage, 265 (34.7%) presented with mild stroke, 236 (30.9%) moderate stroke, and 262 (34.3%) severe stroke, compared with 2,889 (65.3%), 1,071 (24.2%), and 465 (10.5%), respectively, for patients with ischemic stroke. Prestroke physical activity was the strongest predictor of less severe stroke symptoms for intracerebral hemorrhages (aOR 3.57, 99% CI 2.35–5.47, *p* < 0.001) and ischemic strokes (aOR 1.92, 99% CI 1.59–2.33, *p* < 0.001) in the ordinal regression models (Figure 1).

### Poststroke Mortality

**Figure 2:**
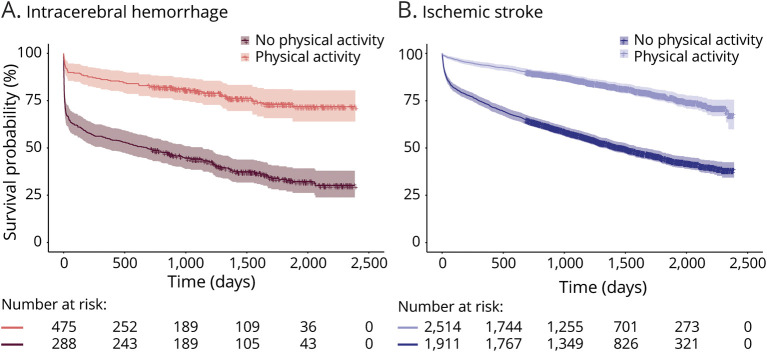
Kaplan-Meier Curves for Poststroke Survival Stratified by Physical Activity Cumulative survival with 99% CIs for (A) patients with intracerebral hemorrhage, and (B) patients with ischemic stroke.

**Figure 3:**
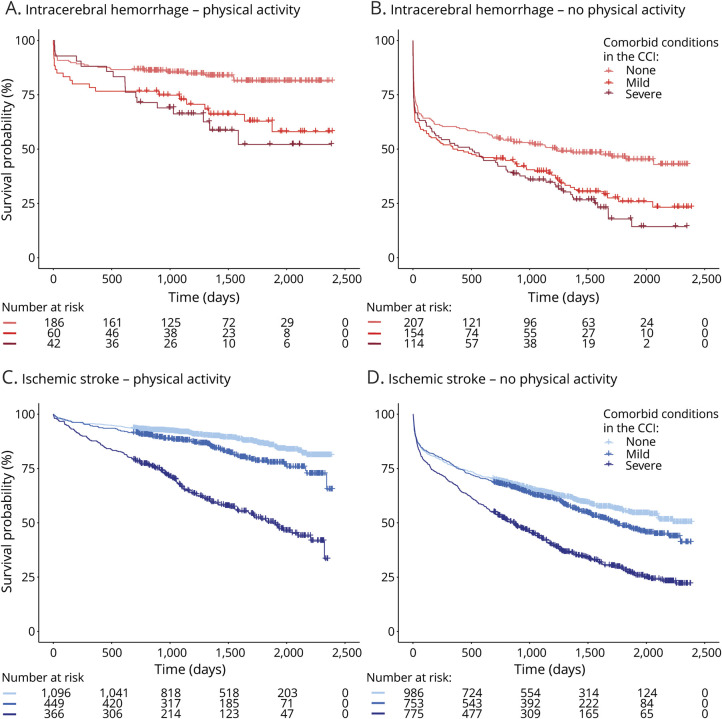
Kaplan-Meier Curves for Poststroke Survival Stratified by Comorbidity Cumulative survival stratified by the weighted CCI as no comorbidity (0 points), mild comorbidity (1–2 points), and severe comorbidity (>2 points) for (A) physically active patients with intracerebral hemorrhage, (B) inactive patients with intracerebral hemorrhage, (C) physically active patients with ischemic stroke, and (D) inactive patients with ischemic stroke. CCI = Charlson comorbidity index.

During a median follow-up of 4.7 (IQR 3.5–5.9) years after the incident stroke, 2,029 (39.1%) of the patients had died (370 [48.5%] after intracerebral hemorrhage and 1,659 [37.5%] after ischemic stroke). After intracerebral hemorrhage, 183 (24.0%) died within 30 days, compared with 329 (7.4%) after ischemic stroke. CVD was the cause of death in 218 (58.9%) patients with intracerebral hemorrhage and in 804 (48.5%) patients with ischemic stroke. Physically active patients had considerably higher survival compared with inactive patients (Figure 2), and the difference remained stable over the course of 5 years (Table 2). Notably, the group of patients with severe comorbidities who were physically active before intracerebral hemorrhage had a higher survival probability compared with inactive patients with no comorbid conditions, and a similar trend was observed for ischemic strokes (Figure 3). The comorbid conditions are summarized in eTable 5 (links.lww.com/WNL/C265).

**Table 2:**
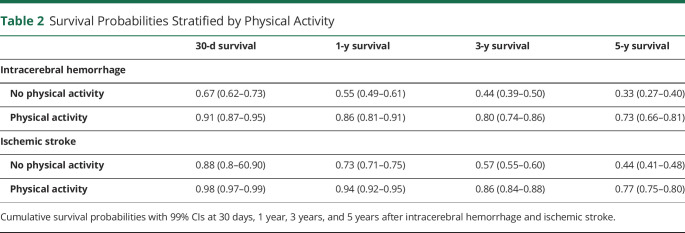
Survival Probabilities Stratified by Physical Activity

Prestroke physical activity was associated with 70% lower short-term mortality after intracerebral hemorrhage (aHR 0.30, 99% CI 0.17–0.54, *p* < 0.001) and 78% after ischemic stroke (aHR 0.22, 99% CI 0.13–0.37, *p* < 0.001) (Table 3). Thereafter was prestroke physical activity associated with a 60% lower risk of long-term mortality after intracerebral hemorrhage (aHR 0.40, 99% CI 0.21–0.77, *p* < 0.001) and 51% after ischemic stroke (aHR 0.49, 99% CI 0.38–0.62, *p* < 0.001). When additionally adjusted for admission stroke severity (NIHSS), prestroke physical activity remained associated with a 52% lower risk of long-term mortality after intracerebral hemorrhage and 48% after ischemic stroke (eTable 6, links.lww.com/WNL/C265).

**Table 3:**
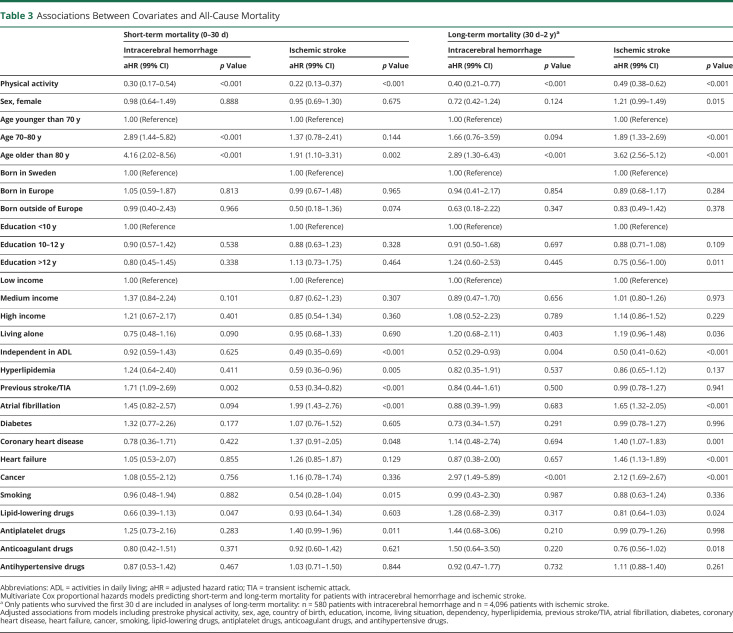
Associations Between Covariates and All-Cause Mortality

### Dose-Response of Physical Activity

There was no statistically significant difference in the odds of less severe stroke symptoms between patients who performed light physical activity ≥4 h/wk and moderate physical activity ≥2 h/wk the year before stroke (Table 4). Moderate prestroke physical activity was associated with a lower crude hazard ratio for poststroke mortality (HR 0.11, 99% CI 0.06–0.19, *p* < 0.001) compared with light physical activity (HR 0.33, 99% CI 0.28–0.39, *p* < 0.001) in patients with ischemic stroke. Although a similar trend was observed for intracerebral hemorrhages, there was no statistically significant difference.

**Table 4:**
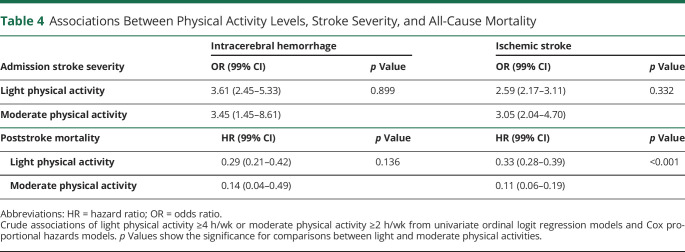
Associations Between Physical Activity Levels, Stroke Severity, and All-Cause Mortality

## Discussion

In this longitudinal, register-based cohort study, we observed that prestroke physical activity was strongly associated with less severe stroke symptoms at hospital admission in patients with intracerebral hemorrhage and ischemic stroke. Notably, the magnitude of this association was greater for intracerebral hemorrhage than for ischemic stroke, with nonoverlapping CIs. Furthermore, we found that prestroke physical activity was independently associated with a lower risk of short-term and long-term all-cause mortality. We also found a dose-response relationship between a higher intensity of prestroke physical activity and reduced mortality hazards after ischemic strokes. However, there were no significant differences between patients with light and moderate physical activities in predictions of stroke severity or poststroke mortality after intracerebral hemorrhage, which is likely contributed by the small proportion of moderately active patients in this group. Although the association between prestroke physical activity and favorable outcomes after stroke has been previously reported, few studies included patients with intracerebral hemorrhage. One previous study has reported separate analyses for intracerebral hemorrhage, in which there was no association between prestroke physical activity and modified Rankin Scale scores at discharge in 221 men with intracerebral hemorrhage from the Physicians' Health Study.^37^

Prestroke physical activity emerged as the strongest predictor of milder admission stroke severity in our study. This finding is consistent with previous research within the PAPSIGOT project, in which patients engaging in light physical activity for ≥4 h/wk were more likely to experience a mild stroke (NIHSS 0–5) compared with those who were less active.^10^ In a larger study, prestroke physical activity for ≥30 minutes, 3 times/week was associated with less severe strokes (NIHSS 1–20) and, particularly, with the lowest quintile of stroke severity (NIHSS 1–5).^12^ Similarly, a dose-dependent relationship between the duration of weekly prestroke physical activity and reduced ischemic stroke severity (NIHSS 0–3) has been previously reported.^7,8^ In another study, prestroke physical activity, defined as >1,000 MET minutes per week, predicted lower NIHSS scores in patients with ischemic stroke.^11^

Independent of admission stroke severity and other risk factors, our findings demonstrate an association between prestroke physical activity and a lower risk of all-cause mortality among patients with intracerebral hemorrhage and ischemic stroke. Comparable results were observed in 2 recent studies with samples including predominantly ischemic stroke cases. Most recently, a higher level of 6-year accumulated prestroke physical activity was associated with lower all-cause mortality and cardiovascular mortality^13^; whereas the other study indicated that inactive individuals, with a basal metabolic rate <1.70, had a greater risk of mortality at 1 and 5 years after stroke.^15^ Earlier, women with no prestroke physical activity were found to have a 39% higher risk of poststroke mortality compared with women with physical activity for >150 min/wk.^14^ Prestroke physical activity has also been previously associated with reduced in-hospital mortality.^12^

Although some studies have failed to detect associations between prestroke physical activity, stroke severity, and poststroke mortality, cumulative evidence suggests that prestroke physical activity improves outcomes after stroke.^20^ Exercise and physical activity are believed to induce brain ischemic tolerance through changes in endothelial function and the inflammatory response, induced angiogenesis and neurogenesis, inhibition of apoptotic pathways, and increased cerebral blood flow.^38^ For intracerebral hemorrhage, the outcome is strongly determined by the volume, localization, and expansion of the hematoma.^39-41^ The role of ischemic tolerance in intracerebral hemorrhage remains unknown; however, a recent preclinical study reported that treadmill exercise before intracerebral hemorrhage in aging mice can decrease hematoma volume, prevent withstanding neurologic deficits, and improve recovery from weight loss.^28^ In addition, physically active individuals are likely to have healthier vessels, with less vascular calcification from atherosclerotic changes, mineral deposition, and cerebral amyloid angiopathy, which may decrease the possibility of hematoma expansion.^42^ Predictors of the outcome after intracerebral hemorrhage may also differ significantly between deep and lobar hematomas.^43,44^ Although hematoma volumes were not analyzed in this study, the location of hematomas was similar between physically active and inactive patients.

The key contribution of this study is the finding of a relationship between prestroke physical activity and favorable outcomes after intracerebral hemorrhage, which has not been previously reported, and is strengthened by the size of the data. This study also draws strength from Sweden's large, comprehensive, and validated inpatient registry data, where underreporting is generally low.^45^ In addition, all persons in Sweden have equal access to tax-funded health care services, and very few with stroke can be expected to not receive hospital care, which increases the generalizability of our results. However, we acknowledge that the limitations of this study make causal inferences impossible. First, the internal validity is limited, due to the observational research design, which is sensitive to several types of bias. It is apparent that the group with prestroke physical activity differs from that with no physical activity in many aspects. Although several potential confounders were adjusted for in the multivariate regression models, there are factors unobserved in this study. In particular, we were not able to adjust for body mass index, which may confound the relationship between prestroke physical activity and stroke outcomes. Second, there is an uncertainty associated with assessments collected from medical records. Third, the use of self-reported retrospective assessments for prestroke physical activity introduces a risk of misclassification and recall bias. The neurologic consequences of stroke may hinder patients from correctly estimating their prestroke activity. However, there is evidence supporting that self-reported physical activity assessments may be reliable and reproducible in stroke populations,^46^ and the SGPALS is a widely used instrument, which has been validated as an indicator of sedentary behavior and cardiovascular risk profile.^47^ In addition, because the data were collected from routine clinical work, all patients and clinicians recording the variables were unaware of our research hypotheses.

Despite the well-known health benefits of physical activity, almost 30% of adults in the world are insufficiently active.^48^ Based on all current knowledge, public health strategies and health care professionals should promote physical activity to decrease the consequences of stroke. Physical activity is considered a safe intervention and can serve as a counterpart to polypharmacy in elderly and cerebrovascular at-risk populations. The results of this study strengthen the notion that a prestroke habit of physical activity may protect the brain in cases of intracerebral hemorrhage and provide new incentives to explore related mechanisms. Future research with prospectively collected data on physical activity is needed to confirm the results of this study.

## Glossary

aHR: adjusted hazard ratio
aOR: adjusted odds ratio
CVD: cerebrovascular disease
IQR: interquartile range
NIHSS: NIH Stroke Scale
PAPSIGOT: physical activity prestroke in Gothenburg
SGPALS: Saltin-Grimby Physical Activity Level Scale
